# Mamba-YOLO-ML: A State-Space Model-Based Approach for Mulberry Leaf Disease Detection

**DOI:** 10.3390/plants14132084

**Published:** 2025-07-07

**Authors:** Chang Yuan, Shicheng Li, Ke Wang, Qinghua Liu, Wentao Li, Weiguo Zhao, Guangyou Guo, Lai Wei

**Affiliations:** 1 School of Computer, Jiangsu University of Science and Technology, Zhenjiang 212100, China; yuanc@stu.just.edu.cn (C.Y.); 241210702113@stu.just.edu.cn (S.L.); 2 School of Automation, Jiangsu University of Science and Technology, Zhenjiang 212100, China; 241110302104@stu.just.edu.cn (K.W.); 241210301214@stu.just.edu.cn (W.L.); 222210301209@stu.just.edu.cn (G.G.); 232210301123@stu.just.edu.cn (L.W.); 3 Jiangsu Key Laboratory of Sericultural Biology and Biotechnology, School of Biotechnology, Jiangsu University of Science and Technology, Zhenjiang 212100, China; wgzsri@126.com; 4 Key Laboratory of Silkworm and Mulberry Genetic Improvement, Ministry of Agriculture and Rural Affairs, The Sericultural Research Institute, Chinese Academy of Agricultural Sciences, Zhenjiang 212100, China

**Keywords:** mulberry leaf disease, Mamba, Haar wavelet, SSM, object detection

## Abstract

Mulberry (*Morus* spp.), as an economically significant crop in sericulture and medicinal applications, faces severe threats to leaf yield and quality from pest and disease infestations. Traditional detection methods relying on chemical pesticides and manual observation prove inefficient and unsustainable. Although computer vision and deep learning technologies offer new solutions, existing models exhibit limitations in natural environments, including low recognition rates for small targets, insufficient computational efficiency, poor adaptability to occlusions, and inability to accurately identify structural features such as leaf veins. We propose Mamba-YOLO-ML, an optimized model addressing three key challenges in vision-based detection: Phase-Modular Design (PMSS) with dual blocks enhancing multi-scale feature representation and SSM selective mechanisms and Mamba Block, Haar wavelet downsampling preserving critical texture details, and Normalized Wasserstein Distance loss improving small-target robustness. Visualization analysis of the detection performance on the test set using GradCAM revealed that the enhanced Mamba-YOLO-ML model demonstrates earlier and more effective focus on characteristic regions of different diseases compared with its predecessor. The improved model achieved superior detection accuracy with 78.2% mAP50 and 59.9% mAP50:95, outperforming YOLO variants and comparable Transformer-based models, establishing new state-of-the-art performance. Its lightweight architecture (5.6 million parameters, 13.4 GFLOPS) maintains compatibility with embedded devices, enabling real-time field deployment. This study provides an extensible technical solution for precision agriculture, facilitating sustainable mulberry cultivation through efficient pest and disease management.

## 1. Introduction

Mulberry (*Morus* spp.), an ancient cultivated plant, can trace its origins back thousands of years to the foothills of the Himalayas in the Asian continent [1,2]. Archaeological evidence and historical records indicate that mulberry cultivation was closely associated with the rise of sericulture, with China initiating the use of mulberry leaves for silkworm rearing and silk production as early as around 2400 BC [3]. Following the opening of the Silk Road, mulberry gradually spread to other regions of Asia, Europe, and Africa, becoming one of the world’s important economic crops [4].

Mulberry leaves serve as the primary feed for silk production, and the global sericulture industry, as a vital sector for textile raw materials, directly depends on the yield and quality of mulberry leaves. The economic value of mulberry leaves extends beyond the silk industry, influencing multiple related fields. Mulberry trees are not only valued for their central role in sericulture but also for the medicinal and nutritional significance of their leaves, fruits, and root bark [5]. Rich in proteins, vitamins, and polyphenolic compounds, mulberry leaves are widely used in functional foods, health supplements, and traditional Chinese medicine [6]. Furthermore, mulberry cultivation positively impacts the ecological environment. With their well-developed root systems, mulberry trees effectively prevent soil erosion and improve soil structure [7]. Therefore, the healthy growth of mulberry trees is crucial not only for the sustainable development of the sericulture industry but also for multiple sectors, including medicine, food, and ecological conservation.

However, the growth of mulberry trees is often threatened by various diseases and pests, which severely affect both the yield and quality of mulberry leaves. In terms of diseases, mulberry anthracnose, brown spot, and bacterial blight are common pathogens during mulberry cultivation. Mulberry anthracnose causes black or brown lesions on leaves, leading to wilting and defoliation in severe cases. Brown spot forms brown necrotic patches on leaves, impairing photosynthesis and nutrient accumulation. Bacterial blight manifests as water-soaked lesions and can result in plant death when infection is severe. Regarding insect pests, the Aulacophora femoralis and Chrysochus are major threats. The Aulacophora femoralis feeds on mulberry leaves, causing leaf deformation, chlorosis, and eventual necrosis. Chrysochus suck plant sap, resulting in yellow speckles and wilting. These pests not only reduce mulberry leaf production but also compromise silk quality, thereby creating ripple effects throughout the sericulture industry and related economic sectors.

Traditional methods for controlling mulberry leaf pests and diseases primarily rely on manual observation followed by chemical pesticide application, yet this approach exhibits several limitations. For instance, manual inspection is inefficient and struggles to achieve large-scale, real-time monitoring. Particularly during the early stages of infestation when symptoms are subtle, cases are frequently missed. In such scenarios, farmers often resort to preventive chemical spraying across entire mulberry fields. However, excessive pesticide use not only fosters pest resistance but also poses significant risks to both environmental safety and human health [8]. With the intensification of global climate change and agricultural practices, the frequency and severity of pest and disease outbreaks have been increasing. The concept of precision agriculture management has gradually gained acceptance, as targeted treatment in smaller areas aligns with both economic benefits for farmers and public health safety. Consequently, efficient and accurate detection and localization of diseased mulberry leaves represent a primary objective for achieving precision agriculture management, as well as a crucial factor in ensuring increased yields in mulberry plantations.

In recent years, with the rapid development of computer vision, artificial intelligence (AI), and machine learning, image analysis and deep learning-based methods for pest and disease detection have gradually become a research hotspot. These technologies enable rapid and accurate identification of plant diseases and pests through automated approaches, providing novel solutions for precision crop health management [9]. In 2022, Li et al. [10] pioneered the application of an improved YOLO algorithm to identify jute diseases, providing a foundation for scientific jute cultivation. Subsequently, in 2023, Lin et al. [11] validated the efficacy and efficiency of computer vision in disease detection by employing UAV-captured images and a Semi-Supervised Contrastive Unpaired Translation Iterative Network for rice blast identification. Both studies demonstrate the potential of computer vision for disease detection in jute and rice, respectively.

Current research methods for mulberry leaf disease detection can be primarily categorized into two approaches: object classification methods and object detection methods. Classification methods focus on identifying disease types but are limited to detecting only a single disease on one leaf at a time, making them incapable of comprehensive diagnosis when multiple diseases co-occur on the same leaf. In contrast, object detection methods advance the capability by achieving precise localization of diseased areas, enabling pixel-level diagnosis for multiple leaves simultaneously.

In the early stages of object classification, disease identification primarily relied on manually extracted features combined with classifiers. Anasuya et al. [12] proposed an automated detection method for mulberry leaf diseases based on image processing and machine learning. By extracting features such as Edge Histogram Descriptors (EHDs), Histogram of Oriented Gradients (HOG), and Gray-Level Co-occurrence Matrix (GLCM), and integrating them with the KNN classification algorithm [13], their approach achieved a high accuracy of 97.5%, pioneering a machine learning solution for precise identification of mulberry leaf diseases. YASIN et al. [14] employed SqueezeNet for deep feature extraction and combined it with a support vector machine (SVM) to classify 10 categories of mulberry leaf diseases, achieving a model accuracy of 77.5%. While these methods demonstrate considerable flexibility in feature extraction and classifier design, their generalization capability in complex backgrounds remains limited.

With the increasing demand for precision agriculture management, improving the accuracy of mulberry leaf disease detection has become a primary objective. Deep learning methods, through their end-to-end learning approach, can automatically extract highly abstract and useful features of mulberry leaf diseases while significantly enhancing target classification accuracy, making them highly sought after by researchers. Duragkar [15] proposed a deep learning-based image classification approach by developing a binary neural network model for mulberry leaf disease detection, which pioneered a new pathway for precise identification of mulberry diseases. Nahiduzzaman et al. [16] proposed a lightweight Parallel Depthwise Separable Convolutional Neural Network (PDS-CNN) for mulberry leaf disease classification, achieving classification accuracies of 95.05% and 96.06% in ternary and binary classification tasks, respectively, while significantly reducing parameters and model size. By incorporating explainable AI (XAI) techniques, this model provides sericulture experts with an efficient and precise tool for mulberry leaf disease identification. Wen et al. [17] proposed an improved mulberry leaf disease recognition method by integrating multi-scale residual networks with Squeeze-and-Excitation Networks (SENets) [18]. Through image enhancement techniques and multi-scale convolutional operations, this approach significantly enhanced model performance, achieving a recognition accuracy of 98.72%, thereby providing an effective technical reference for the intelligent detection of mulberry leaf diseases. Salam et al. [19] employed an improved MobileNetV3Small [20] deep learning model for mulberry leaf disease classification. By incorporating additional convolutional layers and image enhancement techniques, the model’s performance was significantly enhanced. The system achieved over 96% in precision, recall, F1-score, and accuracy metrics. Furthermore, the researchers developed an efficient smartphone application capable of real-time mulberry leaf disease identification.

Compared with object classification, object detection is more suitable for observing the location and severity of mulberry leaf diseases, providing better visualization. Moreover, some object detection algorithms offer models with lower complexity and fewer parameters, making them more applicable for real-time detection on embedded devices and mobile platforms. Currently, object detection algorithms can be broadly categorized into two branches: two-stage detectors and one-stage detectors. Two-stage detectors, such as R-CNN [21] and Faster R-CNN [22], first generate region proposals and then classify and regress them. In contrast, one-stage methods like YOLO [23] and SSD [24] can directly predict object values from an entire image without proposal generation. Since two-stage algorithms require generating region proposals, they incur significant computational overhead, making them generally unsuitable for embedded real-time detection. Therefore, in modern precision agriculture applications, researchers typically prefer one-stage models.Reddy and Deeksha [25] pioneered a convolutional neural network (CNN) and YOLO-based model for mulberry leaf disease detection. Zhang et al. [26] proposed an improved high-precision mulberry leaf disease detection algorithm named YOLOv8-RFMD based on YOLOv8. By incorporating an MDFA block, the algorithm significantly enhanced the detection performance for small lesions, achieving a model mAP50 of 94.3%.

While the aforementioned studies have made significant progress in mulberry leaf disease detection and identification, there remains potential for improvement in both accuracy and speed when detecting multiple targets under natural conditions. Currently, the state-of-the-art (SOTA) in YOLO algorithms is Mamba-YOLO [27], which incorporates the unique architecture of Mamba [28]. Mamba-YOLO integrates the core concepts of Mamba with the YOLO object detection model, leveraging Mamba’s selective mechanism and parallel computing advantages to enhance the model’s adaptability to multi-target detection in complex backgrounds while reducing computational complexity and memory consumption. Experimental results demonstrate that Mamba-YOLO excels in multi-object detection tasks under natural conditions, outperforming traditional YOLO algorithms in both detection accuracy and category recognition capabilities. This provides a new technical direction for real-time detection and precise management of mulberry leaf diseases. Therefore, this study will specifically focus on optimizing the Mamba-YOLO model to further improve its detection accuracy and optimize model size.

## 2. Materials and Methods

### 2.1. Construction of the Dataset

#### 2.1.1. Dataset Acquisition and Annotation

**Figure 1:**
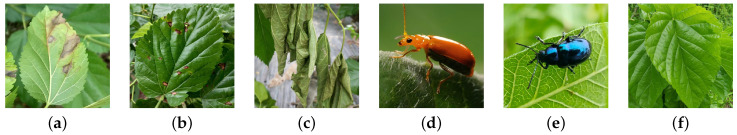
Representative samples of the mulberry dataset: (**a**) mulberry anthracnose, (**b**) mulberry brown spot, (**c**) mulberry bacterial blight, (**d**) aulacophora femoralis, (**e**) Chrysochus, and (**f**) healthy mulberry leaf.

The dataset used in this study was sourced from the publicly available PaddlePaddle dataset. For detailed information, please visit https://aistudio.baidu.com/datasetdetail/265143/0 (accessed on 3 April 2024) to learn more about the "Mulberry Leaf Diseases and Pests Dataset”. The dataset contains six categories of labels: mulberry anthracnose, mulberry brown spot, mulberry bacterial blight, aulacophora femoralis, Chrysochus, and healthy mulberry leaves. In the original dataset, each image contained only sparse annotations with numerous unlabeled targets, which could mislead the model to focus solely on limited regions of interest and cause significant misclassifications. Therefore, comprehensive re-annotation was identified as a critical prerequisite for model training. Consequently, we performed manual relabeling of all 1788 images using LabelImg (version 1.8.6) to demarcate rectangular regions of interest. Representative samples of the annotated images are shown in Figure 1.

#### 2.1.2. Data Augmentation

**Figure 2:**
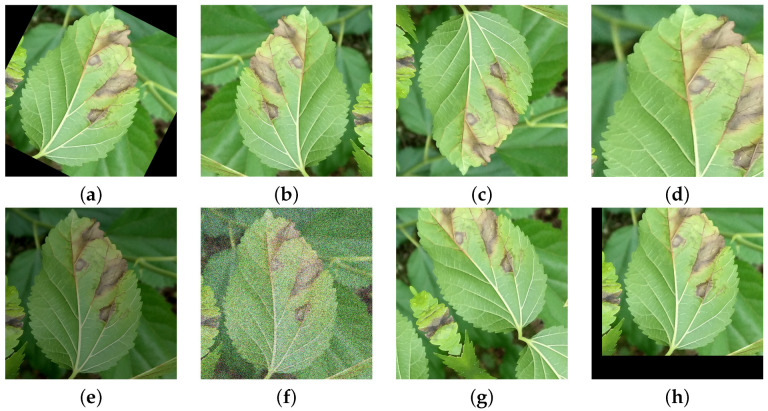
Examples of data augmentation: (**a**) random rotation, (**b**) horizontal flip, (**c**) vertical flip, (**d**) random crop, (**e**) brightness adjustment, (**f**) Gaussian blur, (**g**) random shift, (**h**) random affine transformation.

In the research on mulberry leaf disease detection, image enhancement techniques serve as a crucial approach to address issues including overfitting, insufficient image quantity, and inconsistent image quality caused by factors such as illumination variations, viewing angles, and complex backgrounds. Since deep learning models are prone to overfitting with limited data, image enhancement methods such as random rotation, flipping, and cropping generate diversified samples from finite datasets, thereby improving model generalization. Meanwhile, given the high cost of acquiring annotated data, techniques like brightness adjustment effectively expand the dataset size, alleviating the bottleneck of data scarcity. Furthermore, field-acquired mulberry disease images often suffer from inconsistent quality due to uneven lighting, perspective differences, and cluttered backgrounds. By employing Gaussian noise injection, random translation, and affine transformations, image enhancement simulates environmental variations, enabling models to better adapt to complex scenarios and consequently enhancing detection robustness and accuracy. The original sample is shown in Figure 1a, while the enhanced images are presented in Figure 2.

The detailed categories of the re-annotated dataset and the number of anchor boxes for each category are presented in Table 1. Since performing data augmentation before dataset splitting may lead to data leakage, which would cause model overfitting and adversely affect the model’s overall generalization capability [29], we therefore first randomly divided the dataset into training, testing and validation sets at a 4:1:1 ratio (1189 images for training, 299 images for testing and 300 images for validation), then performed data augmentation on the training set to expand it to 10,701 images. This approach enhances dataset diversity, improves model robustness, and significantly reduces the model’s sensitivity to environmental variations.

### 2.2. Mamba-YOLO Model

Mamba-YOLO is an object detection algorithm that employs a structured state-space sequence model (S4) [30] and Mamba (rooted in state-space models, SSMs) [28] as its backbone architecture, representing one of the newer models in the YOLO family. The SSM framework draws inspiration from Kalman filters and can be conceptualized as a linear time-invariant (LTI) system. This system maps an input sequence u(t)∈R to a continuous output sequence y(t)∈R through an underlying state representation $h\left(t\right)\in R^{N}$. Consequently, the model captures not only the direct input-output relationships but also their temporal dynamics. Mathematically, this system can be described by the following differential equations:

(1) $$h^{\prime }\left(t\right)=Ah\left(t\right)+Bu\left(t\right)$$

(2) y(t)=Ch(t)

where $A\in R^{N\times N}$, $B\in R^{N\times 1}$ and $C\in R^{1\times N}$.

However, in practical scenarios, the processed data is often discrete. Therefore, fixed discretization rules must be employed to discretize the aforementioned equations to make them compatible with deep learning frameworks. Following the Mamba paper, we adopt the Zero-Order Hold (ZOH) method for discretization, which is defined as follows:

(3) $$h^{\prime }\left(t\right)=\bar{A}h_{t-1}+\bar{B}u_{t}$$

(4) $$y_{t}=Ch_{t}$$

where $\bar{A}$ and $\bar{B}$ correspond to the discrete forms of A and B, respectively.

By reformulating the above equation, we obtain a convolutional variant that is more suitable for deep learning implementations. This transformation provides significant parallelization advantages to the model, accelerating both training and inference processes. The mathematical formulation is defined as follows:

(5) $$\bar{K}=\left(C\bar{B},C\bar{A}\bar{B},\ldots ,C{\bar{A}}^{L-1}\bar{B}\right)$$

(6) $$y=u*\bar{K}$$

where $\bar{K}\in R^{K}$ denotes the structured convolution kernel after transformation, with L representing the input sequence length.

**Figure 3:**
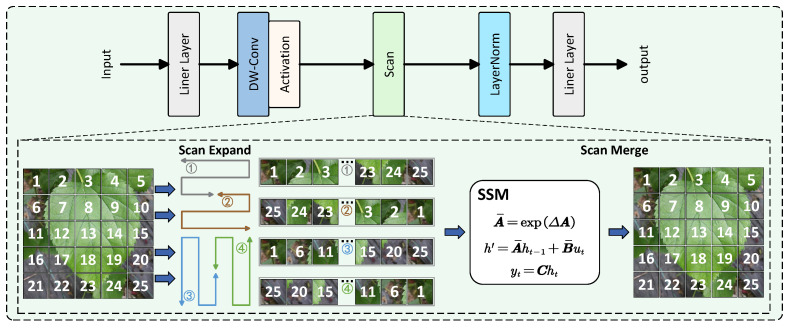
Structural diagram of the SS2D (2D-Selective-Scan for Vision Data) Block, illustrating the cross-scanning, selective scanning, and cross-merging operations.

VMamba [31], as one of the latest object detection models based on the Mamba architecture for computer vision, incorporates a 2D-Selective-Scan for Vision Data (SS2D) mechanism that draws inspiration from Mamba’s selective scanning approach in language processing, making it more suitable for 2D image analysis. The SS2D algorithm consists of three key steps: cross-scanning, selective scanning (filtering), and cross-merging. For input image data, SS2D first unfolds image patches sequentially along four directional paths. The SSM model then selectively processes these four unfolded data streams before reconstructing the outputs along their original scanning directions to generate the final result. By employing a center-focused path unfolding strategy, the SS2D algorithm enables each pixel to establish relationships with all other pixels, achieving a global receptive field similar to Transformer architectures while maintaining significantly fewer parameters and lower computational costs. The structural diagram of the SS2D Block is illustrated in Figure 3.

**Figure 4:**
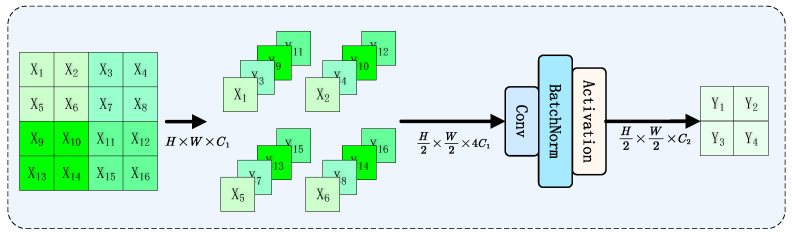
Vision Clue Merge downsampling block architecture, which reduces computational costs through channel stacking and 1×1 convolutions, operating similarly to dilated convolution with a dilation rate of 2.

The Mamba-YOLO [27] model introduces several key improvements over the original VMamba architecture. First, it incorporates a Vision Clue Merge downsampling block (as shown in the Figure 4), which operates similarly to a dilated convolution with a dilation rate of 2, but significantly reduces computational costs through channel stacking followed by 1×1 convolutions. Additionally, the model enhances the VSSBlock of VMamba by introducing two novel components: the LS Block (LocalSpatial Block) and RG Block (ResGated Block). The LSBlock expands the local receptive field through a depth-wise convolution followed by an MLP, enabling the Mamba architecture to better capture long-range spatial dependencies. The RGBlock effectively preserves spatial structural information while enhancing global feature extraction capabilities by combining gating mechanisms, residual connections, and depthwise separable convolutions, all achieved with minimal computational overhead. These innovations collectively lead to substantial improvements in both the model’s representational capacity and overall performance.

### 2.3. Mamba-YOLO-ML Model

**Figure 5:**
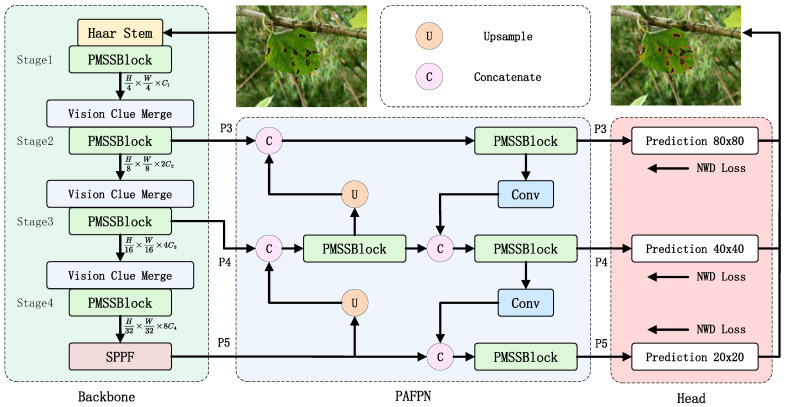
Illustration of Mamba-YOLO-ML architecture.

This study aims to adjust the model architecture without compromising accuracy to achieve a smaller parameter size and lower computational complexity, making it more suitable for embedded devices. Special adaptations were made for mulberry leaf disease detection, such as enhanced focus on leaf texture features and minute characteristics. Accordingly, we named this optimized model for mulberry leaf disease detection as Mamba-YOLO-ML (Mamba-YOLO for Mulberry Leaf Disease). The improved network architecture is illustrated in Figure 5, with the specific modifications detailed below:

- A Phase-Modular Design approach was adopted to redesign the ODSS Block in Mamba-YOLO into the PMSS Block (Phase-Modular SSM Structured Block), which better leverages the SSM architecture to enhance both global and local receptive fields. Specifically, the FG Block (Feature Gated Block) replaces the original RG Block in the MLP stage following SS2D, improving the model’s capability to filter pixel-level, spatial, and channel dimensions, ultimately extracting more discriminative feature maps. Meanwhile, the CE Block (Content Enhancement Block) substitutes the original LS Block to strengthen data details before input into the SSM algorithm.
- Inspired by Haar wavelet downsampling [32], a more lightweight Haar Stem block was designed to replace the original Simple Stem in the model.
- The original CIOU loss function was replaced with the NWD (Normalized Wasserstein Distance) loss function [33], which demonstrates lower sensitivity to geometric deviations in small targets.

#### 2.3.1. PMSS Block

The architecture of YOLO primarily consists of three components: the backbone network, the path aggregation feature pyramid network (PAFPN), and the detection head. Input data first passes through four hierarchical stages (Stage 1–4) in the backbone network. Subsequently, the PAFPN and detection head receive feature maps from the last three stages of the backbone as inputs to generate the final output.

**Figure 6:**
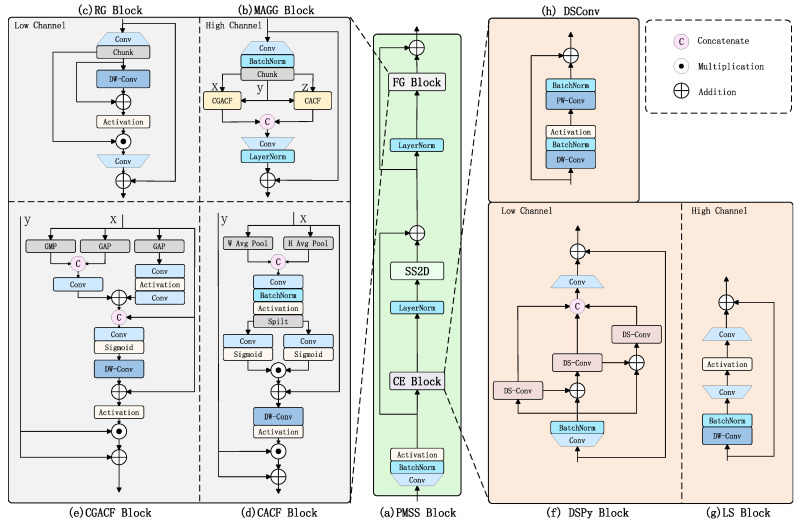
Illustration of PMSS Block architecture.

The original Mamba-YOLO model algorithm, within its core ODSS Block, processes data through SS2D screening to reach high-channel stages (i.e., after the first stage of the backbone network). At this point, each pixel possesses a relatively large receptive field, and the abstracted information from these receptive fields is stored in their corresponding channels. Consequently, the original RG Block, which has an insufficiently complex structure and fails to fully leverage channel attention, exhibits a negative impact on the responsibilities of the MLP. In contrast, before SS2D screening is applied in the low-channel stage (i.e., the first stage of the backbone network), the data undergoes only two convolutional layers, resulting in smaller receptive fields for each pixel and a lack of complex abstract information. The LS Block used in the original model employs only a single depthwise (DW) convolution to enhance the relevant receptive fields, which means that when the SS2D Block performs screening, the associated local receptive fields remain too small. To address these issues, we designed the PMSS Block to replace the original ODSS Block. Within this new block, we propose two novel modules—the CE Block and the FG Block—to improve how feature maps are enhanced across different channel dimensions. The overall structure of the PMSS Block is illustrated in Figure 6.

Research has demonstrated that the Mamba block based on the State-Space Model (SSM) mechanism exhibits global receptive field capabilities comparable to those of Transformer architectures [28,31]. However, similar to Transformers, its parameter learning process remains computationally intensive. We propose that preprocessing the input data to facilitate feature extraction prior to entering the Mamba block could significantly accelerate its parameter learning. To achieve this, we designed the CE Block, which consists of two components: the DSPy Block enhances feature representation in low-channel scenarios, while the LS Block (from Mamba-YOLO) captures local features in high-channel spaces while simultaneously reducing computational costs and parameter quantities.

The DSPy Block, as shown in Figure 6f, adopts the concept of pyramid convolution [34] to enhance the model’s initial receptive field by extracting multi-scale features. To reduce the model’s parameter count and computational complexity, we employ a combination of depthwise convolution and pointwise convolution from MobileNet [20], supplemented with residual connections, forming depthwise separable convolution (DSConv) as shown in Figure 6h to replace traditional convolution. To strengthen the receptive field under low-channel conditions while preserving local information as much as possible, the designed DSPy Block references the CSP [35] architecture by splitting channels into three parts. Three DSConv layers with kernel sizes of 1, 3, and 5 are arranged in a pyramidal sequence to form depthwise separable pyramid convolution. Both ends of this structure are enhanced with 1×1 convolutional MLP layers to improve robustness, and the entire module utilizes residual connections to mitigate model overfitting. For high-channel scenarios where each pixel possesses sufficiently rich channel information and adequate receptive field coverage, we retain the functionality of the LS Block, as shown in Figure 6g.

The incorporation of attention mechanisms followed by nonlinear layers or FFN (Feed-Forward Network) has proven to be an effective design for enhancing model accuracy [31,36,37]. Through unidirectional information flow and nonlinear transformations, this approach assists the model in extracting features from complex data, particularly when high-dimensional features are not easily separable. Therefore, we designed an FFN-like block called the FG Block, which consists of distinct components for processing low- and high-channel data. For low-channel data, which is more amenable to aggregation and separation, we employ the structurally simpler RG Block. Conversely, for highly abstract high-channel data that cannot be effectively separated using simple feature extraction methods, we utilize the MAGG Block, as illustrated in Figure 6a.

Multi-Attention Guided Gated Block (MAGG Block) integrates Coordinate Attention [38], Content-guided Attention (CGA) [39], and a dynamic gating mechanism to achieve adaptive filtering of feature maps across channel dimensions, spatial dimensions, and pixel-level feature weights in high-channel scenarios. Since the original CA Block and CGA Block only perform attention filtering on individual feature maps and cannot be effectively applied to gating mechanisms, we designed dedicated gating blocks for dual inputs: CACF (Coordinate Attention for Couple Feature) and CGACF (Content-guided Attention for Couple Feature), as illustrated in Figure 6d,e. The MAGG Block first splits the input features into three branches via a 1×1 convolutional fully connected layer. Two branches, respectively, undergo CACF (fusing Coordinate Attention) and CGACF (Content-guided Attention) computations to generate two gated feature maps. These gated maps then filter information from the third feature branch, producing two pathway feature maps. Finally, the two feature streams are restored to the input channel dimension through another 1×1 convolutional fully connected layer and combined with the input via a residual structure. For low-channel feature maps, the SS2D block exhibits slower and less pronounced processing when handling relatively specific features that have not yet developed a high receptive field. Therefore, the RG Block as shown in Figure 6c is retained for processing low-channel feature maps to ensure effective feature extraction.

To address the issue of defining high and low channels and rationally arranging module distribution in the PMSS Block, we designed a PMSS Block stage allocation table (Table 2). Notably, in the PAFPN stage (which was not discussed in the previous sections), we continue to employ both the MAGG Block and DSPy Block as the two modules of the PMSS Block, since the channel dimension here remains greater than or equal to that of Stage4.

#### 2.3.2. Haar Stem Block

**Figure 7:**
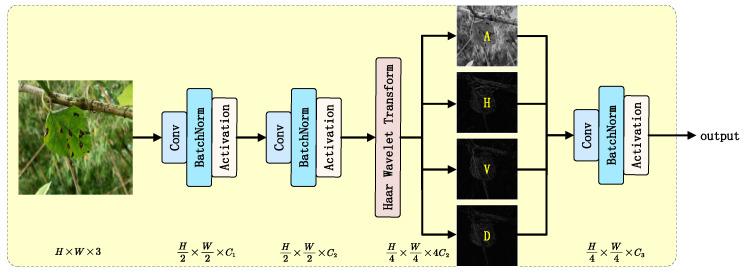
Architecture of the Haar Stem block.

In agricultural monitoring scenarios, the morphological characteristics of mulberry leaves (e.g., vein distribution, margin curvature, and local textures) are crucial for target detection accuracy. Traditional convolutional neural networks typically employ strided convolution or pooling operations for spatial downsampling during initial feature extraction. While these operations reduce computational complexity, they often lead to the loss of high-frequency detail information, resulting in insufficient fine-grained feature representation. To address the need for preserving complex vein textures and disease features in mulberry leaf images, this study proposes a Haar wavelet transform-based feature downsampling block called Haar Stem, as illustrated in Figure 7. The core concept combines Haar wavelet transform with learnable convolutions to achieve information preservation and feature enhancement through multi-resolution analysis. While reducing spatial resolution, this block encodes high-frequency details into channel dimensions, providing subsequent network layers with more discriminative features.

The Haar wavelet transform is an orthogonal, compactly supported multi-scale analysis tool widely used in image coding, edge extraction, and binary logic design [40]. It achieves lossless downsampling by decomposing signals into low-frequency approximation components (A) and high-frequency detail components (H, V, D). The scaling function ϕ(x) and wavelet function ψ(x) of the one-dimensional Haar transform are mathematically defined as follows:

(7) $$\phi \left(x\right)=\left\{\begin{array}{cc} 1, & 0\leq x<1\\ 0, & \mathrm{otherwise} \end{array}\right.$$

(8) ψ(x)=ϕ(2x)−ϕ(2x−1)

For the two-dimensional input feature map $X\in R^{H\times W\times C}$, row–column separable filtering is performed to generate four components:

(9) Haar(X)={A,H,V,D}

Here, $A\in R^{\frac{H}{2}\times \frac{W}{2}\times C}$ represents the low-pass filtered approximation component, which preserves the global structure, while $H,V,D\in R^{\frac{H}{2}\times \frac{W}{2}\times C}$ denote the high-frequency detail components in the horizontal, vertical, and diagonal directions, respectively, capturing edge and texture information.

By concatenating the four components transformed by the Haar wavelet along the channel dimension, the final output $Y\in R^{\frac{H}{2}\times \frac{W}{2}\times 4C}$ is obtained, which has four times the number of channels compared with the original input while reducing the spatial resolution to one-fourth of the original. Compared with traditional compensation convolutions, the Haar wavelet transformation does not increase model parameters and slightly reduces computational costs, while still achieving lossless encoding of spatial information into the channel dimension.

#### 2.3.3. NWD Loss Function

In the context of mulberry leaf pest and disease monitoring, object detection faces two core challenges: first, targets such as disease spots and pests on mulberry leaves exhibit notably small-scale characteristics (average pixel area <32×32), making the CIoU metric in Mamba-YOLO highly sensitive to positional deviations of tiny objects; second, dense occlusion phenomena in mulberry canopy images cause bounding box IOU loss functions to be easily disturbed by local overlap noise. To address these issues, this study introduces the Normalized Wasserstein Distance (NWD) [33] as a core component of the bounding box regression loss function. The theoretical advantage of this approach lies in its modeling of bounding boxes as 2D Gaussian distributions and utilization of Wasserstein distance to measure inter-distribution similarity, which demonstrates stronger robustness against geometric deviations of small targets.

The Wasserstein distance, as a core metric in optimal transport theory, effectively measures the discrepancy between two probability distributions. For the 2D object detection problem, given a predicted bounding box $B_{p}=\left(x_{p},y_{p},w_{p},h_{p}\right)$ and a ground truth bounding box $B_{g}=\left(x_{g},y_{g},w_{g},h_{g}\right)$, we first convert them into 2D Gaussian distribution representations:

(10) $$N_{p}=N\left(\mu_{p},\Sigma_{p}\right)=N\left(\left[\begin{array}{c} x_{p}\\ y_{p} \end{array}\right],\left[\begin{array}{cc} \frac{w_{p}^{2}}{4} & 0\\ 0 & \frac{h_{p}^{2}}{4} \end{array}\right]\right)$$

(11) $$N_{g}=N\left(\mu_{g},\Sigma_{g}\right)=N\left(\left[\begin{array}{c} x_{g}\\ y_{g} \end{array}\right],\left[\begin{array}{cc} \frac{w_{g}^{2}}{4} & 0\\ 0 & \frac{h_{g}^{2}}{4} \end{array}\right]\right)$$

The diagonal elements of the covariance matrix reflect the scale characteristics of the bounding boxes. According to the closed-form solution formula for the Wasserstein distance between Gaussian distributions:

(12) $$W^{2}\left(N_{p},N_{g}\right)=\left|\right|\mu_{p}-\mu_{g}{\left|\right|}^{2}+Tr\left(\Sigma_{p}+\Sigma_{g}-2{\left(\Sigma_{p}^{1/2}\Sigma_{g}\Sigma_{p}^{1/2}\right)}^{1/2}\right)$$

To mitigate the impact of scale variations in targets on distance metrics, a scale normalization coefficient *C* is introduced to construct the Normalized Wasserstein Distance:

(13) $$NWD\left(N_{p},N_{g}\right)=\exp\left(-\frac{\sqrt{W_{2}^{2}\left(N_{A},N_{B}\right)}}{C}\right)$$

Here, the constant *C* is related to the data characteristics. In this experiment, we set C=12.8 to maintain dimensional consistency. The NWD metric is constrained to the range (0, 1], sharing similar semantics with IoU but exhibiting smoother gradient properties. When the predicted bounding box perfectly aligns with the ground truth box, NWD = 1; as the offset increases, NWD decays exponentially. The final NWD loss function is defined as

(14) $$L_{NWD}=1-NWD\left(N_{p},N_{g}\right)$$

The loss function is continuously differentiable within the (0, 1] interval and exhibits smoother gradient variations compared with IoU Loss in small-target detection scenarios, which facilitates more stable model convergence.

### 2.4. Test Platform

The experiments were conducted on a computing platform equipped with an NVIDIA RTX4070 GPU (12GB VRAM) for accelerated computation, paired with an Intel 13600KF processor (3.5GHz base frequency) and 32GB DDR5 RAM. The software environment was built upon the PyTorch 2.1 framework with CUDA 12.3 providing underlying parallel computing support.

The input image resolution was standardized to 640×640×3 pixels with a fixed batch size of 8. The dataset underwent processing through Mosaic [41] and Mixup [42] data augmentation strategies. The optimizer employed was Stochastic Gradient Descent (SGD) with an initial learning rate of 0.01, dynamically adjusted using a Cosine Annealing strategy. The momentum parameter was set to 0.937 with a weight decay coefficient of 0.0005. The training procedure spanned 300 epochs, incorporating a 3-epoch warm-up phase to mitigate gradient oscillation.

### 2.5. Evaluation Metrics

The performance evaluation of object detection algorithms requires quantitative analysis from multiple dimensions. This section systematically discusses the improved YOLO algorithm based on key metrics, including precision, recall, mean average precision (mAP), model parameter size, model size, and computational complexity.

Precision (P) reflects the proportion of true positive samples among all samples predicted as positive by the model, calculated as

(15) $$P=\frac{TP}{TP+FP}$$

where TP (True Positive) represents the number of correctly detected positive samples, while FP (False Positive) denotes the number of misidentified negative samples.

Recall (R) measures the model’s ability to cover true positive samples, defined as

(16) $$R=\frac{TP}{TP+FN}$$

where FN (False Negative) indicates the number of undetected positive samples.

The mean average precision (mAP) comprehensively evaluates the precision–recall curves across different confidence thresholds, serving as a core evaluation metric for object detection. The mAP is obtained by calculating the arithmetic mean of average precision (AP) across all categories, with its mathematical expression being

(17) $$AP=\int_{0}^{1}P\left(R\right)dR$$

(18) $$mAP=\frac{1}{N}\sum_{i=1}^{N}AP_{i}$$

Here, N represents the total number of categories, and AP is calculated by interpolating the area under the PR curve for the i-th category. In early literature, mAP specifically denoted the value at a 50% IoU threshold (mAP50). The COCO dataset [43] further introduced mAP50:95, which represents the average mAP across IoU thresholds ranging from 0.5 to 0.95 with a step size of 0.05, providing a comprehensive evaluation of the model’s localization accuracy.

## 3. Results

### 3.1. Model Performance Comparison

**Figure 8:**
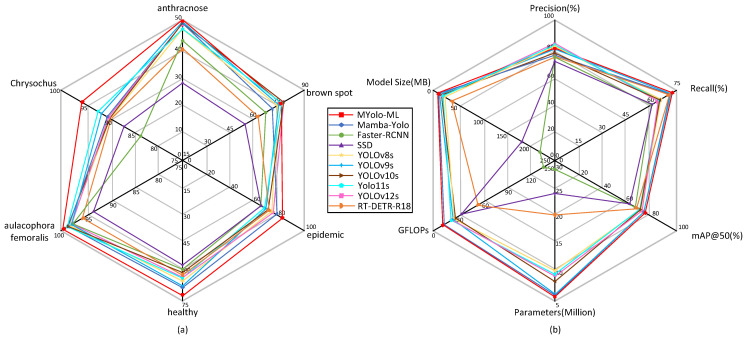
Radar charts (**a**) demonstrating the mAP values of each model for different pest/disease categories and (**b**) illustrating the performance metrics of each model.

To comprehensively evaluate the detection performance of the improved model (Mamba-Yolo-ML), this study conducted comparative experiments with various mainstream object detection algorithms, including traditional two-stage models (Faster R-CNN [22], SSD [24]), YOLO series (YOLOv8, YOLOv9 [44], YOLOv10 [45], YOLOv11 [46], and the recently updated YOLOv12 [47]), as well as Transformer-based architectures (RT-DETR [48]). The two-stage models were implemented using the mmdetection [49] library, while the YOLO series and RT-DETR were based on the ultralytics library. All models were trained and tested under identical experimental conditions. Table 3 and Figure 8b present comparative results of the models in terms of precision, recall, mAP50, mAP50:95, number of parameters, GFLOPs, and model size. Figure 8a illustrates the mAP50 values for each category detected by the different models.

In terms of detection accuracy, Mamba-Yolo-ML outperforms traditional two-stage models with a Precision of 80.7% and a Recall of 72.1%. Its mAP50 (78.2%) and mAP50:95 (59.9%) show improvements of 1.1% each compared with the original Mamba-YOLO. When compared with the latest variants in the YOLO series, our model demonstrates varying degrees of superiority across all evaluation metrics. Notably, many comparative YOLO models exhibit the phenomenon of high precision but low recall, indicating their limited detection coverage and difficulty in identifying occluded ground truths. In species-specific detection tasks, our model only underperforms YOLOv10-12s in the brown spot category, while demonstrating significantly better performance than other models across all other pest categories. Benefiting from the selective scanning mechanism of the SSM architecture, which handles long-range dependencies with linear complexity, SSM-based models achieve global receptive fields and consequently higher detection accuracy. Since Transformer architectures require extensive training to achieve stable convergence [37]; the SSM architecture proves to be a superior choice when working with smaller datasets.

From the perspective of computational efficiency, Mamba-Yolo-ML achieves a GFLOPS of 13.4, representing a 1.5% reduction compared with the original Mamba-Yolo (13.6 GFLOPS). This performance significantly surpasses two-stage models and complex architectures based on Transformer frameworks (e.g., RT-DETR), while also maintaining the lowest computational cost among comparable small-scale YOLO variants. Regarding model parameters, Transformer-based architectures require storing massive weight matrices ($W_{Q},W_{K},W_{V}$), resulting in substantial memory footprint for real-time inference models. In contrast, Mamba-Yolo-ML achieves the highest mAP (59.9%), with only 5.6 M parameters and 11.1 MB storage size, demonstrating inherent advantages for storage- and memory-constrained devices such as embedded systems.

### 3.2. Analysis of Detection Visualization Results Across Different Models

According to Table 3, both Faster R-CNN and SSD models exhibit not only lower mAP but also larger model sizes and higher GFLOPs compared with the YOLO series, making them unsuitable for mulberry leaf disease detection and deployment on mobile embedded devices. YOLOv9s and YOLOv12s demonstrate superior accuracy among newer YOLO series models, showing performance closest to our proposed Mamba-Yolo-ML model, thus serving as more appropriate comparative benchmarks. To further investigate Transformer-based architectures, we selected Mamba-Yolo-ML, Mamba-Yolo, YOLOv9s, and YOLOv12s as our detection models for identifying various types of mulberry leaf diseases and pests.

This visualization comparison experiment primarily demonstrates the model’s accuracy in detecting small targets, heavily occluded objects, and disease textures with slender features. In the visualized results, red represents anthracnose, pink indicates brown spot, orange denotes bacterial blight, yellow signifies healthy leaves, cyan represents Aulacophora femoralis, and green represents Chrysochus. Due to the limited selection of experimental images, not all of these categories may be present in the results.

**Figure 9:**
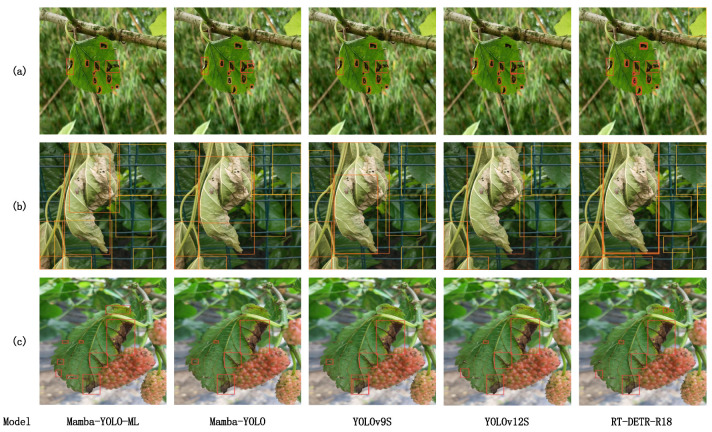
Visualization of detection results across different models: (**a**) small targets, (**b**) detection under occlusion, (**c**) disease textures with slender features.

Figure 9 demonstrates the detection results of different models on several sample images. In the case of mulberry anthracnose lesions presented in Figure 9a, Mamba-YOLO-ML successfully detected all diseased areas with precise bounding boxes, while Mamba-YOLO, YOLOv9s, YOLOv12s, and RT-DETR exhibited significant shortcomings in extracting small target features—all failed to identify the small anthracnose lesion in the lower right corner of the mulberry leaf. Notably, Mamba-YOLO produced false detections. Both Mamba-YOLO and RT-DETR showed bounding box overlap issues, where RT-DETR’s problem might stem from the Transformer’s self-attention mechanism over-focusing on certain salient regions, causing query vectors to generate similar predictions for those areas. For Mamba-YOLO, this issue likely originates from the gating mechanism in SS2D Block failing to properly gate non-redundant feature maps and inadequate enhancement of detail features in low channels.

In the overlapping detection case of mulberry bacterial blight shown in Figure 9b, YOLOv12s and RT-DETR performed poorly, failing to completely detect all four diseased leaves in the image—both models mistakenly identified two centrally located bacterial blight leaves as a single instance. Although YOLOv9s detected the two central diseased leaves, its prediction boxes showed significant deviation from the ground truth. Benefiting from the superior selective properties of the SSM architecture, Mamba-YOLO completely detected the two central bacterial blight leaves. Moreover, with wavelet transform separating high/low-frequency features to distinctly isolate leaf contours and FG Block’s multi-attention gating mechanism (pixel-wise, channel-wise, and global attention) in high channels, Mamba-YOLO-ML demonstrated enhanced capability to distinguish between similar objects, resulting in prediction boxes closer to ground truth.

Regarding vein texture disease detection in Figure 9b, only Mamba-YOLO-ML successfully identified the slender diseased vein pattern. This superior performance likely stems from the model’s Stem stage employing Haar wavelet transform to separate high/low-frequency information, preserving all textural details in the image. This processing makes the model more sensitive to morphological features like vein textures and improves prediction accuracy.

### 3.3. Ablation Study

#### 3.3.1. Ablation on Mamba-Yolo-ML

To validate the independent contributions and synergistic effects of the improved blocks on model performance, we conducted ablation experiments. Table 4 presents the results of these ablation studies. The detailed analysis of each block is as follows.

The baseline model (Mamba-YOLO) achieved 77.1% in mAP50, with significant performance improvements observed after introducing various enhancement blocks. When the CE block was added individually, Precision increased by 0.9%, Recall improved by 1.1%, and mAP50 rose by 1.0%. The CE block enhances local receptive fields under low-channel conditions through depthwise separable pyramid convolution, significantly improving the extraction capability of small lesion features. However, GFLOPS increased from 13.6 to 14.2, indicating a slight rise in computational complexity.

Upon further integrating the FG block (complete PMSS Block structure), the model achieved 80.9% Precision, 71.9% Recall, and 78.0% mAP50. The FG block employs a multi-dimensional attention mechanism to filter redundant features in high-channel scenarios while boosting classification accuracy. It simultaneously compresses the parameter size to 5.6 M, demonstrating the effectiveness of its lightweight design.

When Haar wavelet downsampling was applied independently, mAP50:95 increased by 0.6%, with GFLOPS slightly decreasing to 13.5. By encoding spatial high-frequency information into channel dimensions through wavelet transform, the model retains small-level vein texture details, even during 4× downsampling, which is crucial for early-stage lesion detection.

Replacing CIoU with NWD led to a substantial 5.6% improvement in Precision but a 3.5% decline in Recall. NWD models bounding boxes via Gaussian distribution, alleviating geometric deviation sensitivity for small targets and significantly reducing False Positives (e.g., background misclassification rate decreased by 3%). However, its stringent metrics for tiny objects may cause some low-confidence positive samples to be missed, requiring further threshold optimization to balance precision and recall.

The CE + FG combination reduced model parameters by 6.7% and GFLOPS by 1.5%, while increasing mAP50:95 by 0.5%. This demonstrates that the PMSS Block achieves a balance between computational efficiency and detection accuracy through its phased feature processing strategy. The fully improved model (PMSS Block + Haar Stem + NWD) delivered optimal overall performance (mAP50:95 = 59.9%, Precision = 80.7%, Recall = 72.1%), with both parameter count and computational costs lower than the baseline model, indicating an effective trade-off between accuracy and efficiency in the proposed enhancements.

#### 3.3.2. Ablation on DSPy Block

**Figure 10:**
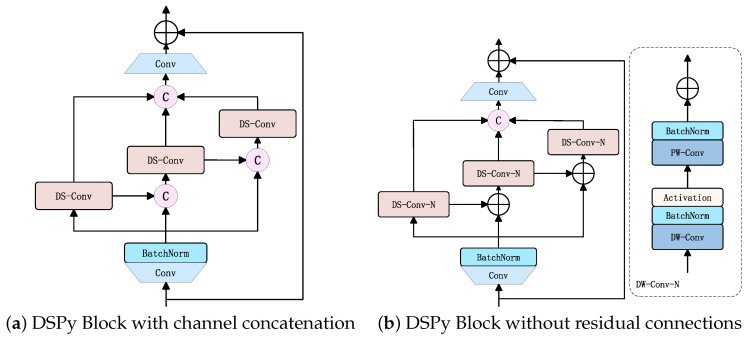
DSPy Block variants.

To validate the accuracy and efficiency of the DSPy Block, as shown in Figure 6f, in highly abstracted mapping of mulberry leaf disease symptoms and pest information under low-channel conditions, as well as its capability to capture pixel-level local dependencies, we designed two additional comparative experiments:

- Replacing channel-wise addition with channel concatenation in the DSPy Block structure, as illustrated in Figure 10a.
- Removing residual connections from the depthwise separable pyramid convolution, as depicted in Figure 10b.

The experimental results of these modifications alongside the original DSPy Block are presented in the Table 5. Notably, while the channel concatenation strategy improved precision, it substantially increased model parameters and computational costs compared with other enhancement approaches, highlighting the need for careful trade-offs between accuracy gains and practical resource constraints. Upon removing residual connections, we observed improvements in Precision and mAP, but a significant decline in Recall, underscoring the critical role of residual connections in preserving feature integrity. Although the DSPy Block exhibited minor performance gaps in certain metrics relative to other variants, its superior overall performance—particularly in robustness and efficiency under complex scenarios—demonstrates its practical feasibility for real-world applications.

#### 3.3.3. Ablation on MAGG Block

**Figure 11:**
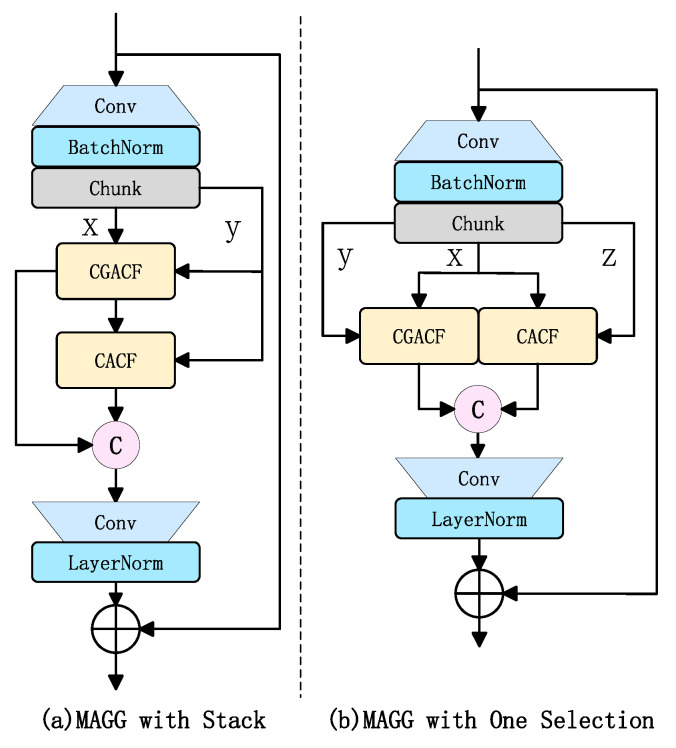
MAGG Block variants.

To validate the effectiveness of the MAGG Block, as shown in Figure 6b, in addressing the SSM sequence model’s sensitivity to data receptive fields and weak image localization in high-channel scenarios, as well as its contribution to model lightweighting, we designed multiple comparative experiments:

- Based on the MAGG Block, the CGACF and CACF blocks are stacked for use, as shown in Figure 11a.
- Building upon the MAGG Block, one channel from the three channels is simultaneously allocated to both the CGACF and CACF blocks to serve as the gating mechanism for the other two channels, as illustrated in Figure 11b.

The ablation study results (as shown in Table 6) indicate that the stacked CGA and CA blocks approach led to a 0.4% decrease in mAP50, while other metrics remained comparable to the baseline model. Using a single channel as a dual-gate mechanism significantly increases Precision but sharply decreases Recall, suggesting that while single-channel gating enhances local feature selection, excessive channel compression leads to spatial information loss. Ultimately, we opted to retain the MAGG Block instead of the stacking approach, as the latter performed suboptimally in ablation studies for Mamba-Yolo-ML. This may be attributed to potential feature interaction redundancy caused by block stacking, which fails to effectively utilize multi-dimensional channel information.

#### 3.3.4. Ablation on Haar Wavelet Position

The application effects of Haar wavelet transform at different levels of the improved Mamba-YOLO are shown in Table 7. Its deployment strategy during the feature extraction stage significantly impacts both the performance balance and computational efficiency of the mulberry pest and disease detection model. When implementing Haar transform at the Stem layer, the model effectively preserves low-frequency global features of mulberry leaf textures through front-loaded frequency-domain decomposition, achieving an mAP50:95 of 59.4%—a 0.6% improvement over downsampling layer deployment—while reducing GFLOPS by 0.1. This demonstrates the strategy’s superior compatibility with the gradual transition characteristics between diseased and healthy tissues in agricultural imagery, owing to its early-stage suppression of high-frequency noise. Although Haar deployment at downsampling layers yields marginally higher Recall, the concurrent decline in both mAP50 (77.3%) and detection accuracy (76.4%) exposes the risk of excessive high-frequency detail loss in deep feature spaces, potentially compromising the model’s discriminative capability for small targets like disease spots and pests.

#### 3.3.5. Ablation on Loss Function

For the task of mulberry leaf pest and disease detection characterized by diverse target scales, irregular morphologies, and numerous small objects, this study systematically evaluated the optimization mechanisms and adaptability differences of various bounding box loss functions, with results presented in Table 8. Experimental results demonstrate that while CIOU [50] loss improves localization accuracy (Precision 80.9%) over basic IOU by introducing aspect ratio constraints, its ambiguous definition of aspect ratios may lead to regression deviations for complex morphological symptoms. EIOU [51] achieves a balance between detection accuracy (Precision 79.4%) and convergence efficiency by explicitly decoupling width-height differences and incorporating Focal Loss [52] to address sample imbalance, yet its reduced recall rate (69.7%) reveals insufficient sensitivity to small-scale lesion features.

Although SIOU [53] loss employs an angle penalty mechanism for rapid axis alignment and slightly outperforms CIOU in mAP50:95 (59.4%), its freedom constraints may weaken the model’s adaptability to non-axial distributions of mulberry leaf diseases, resulting in limited overall detection performance (mAP50 77.0%). WIOU [54] loss optimizes sample quality imbalance through dynamic weight allocation but exhibits weaker representation capability for boundary-ambiguous targets (mAP50 77.0%).

Notably, NWD loss [33] abandons traditional geometric measurement paradigms and instead models the global similarity between predicted boxes and ground truth distributions using Wasserstein distance. While maintaining high precision (Precision 80.7%), it significantly improves Recall (72.1%) and multi-scale generalization capability (mAP50:95 59.9%). This is attributed to its strong robustness against shape distortions, partial occlusions, and small targets.

### 3.4. Detection on Mobile Devices

**Figure 12:**
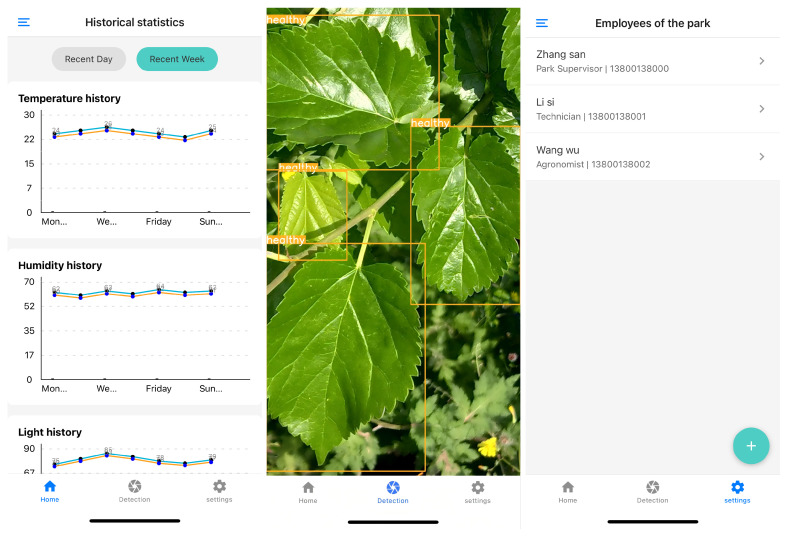
Screenshots of the mulberry farm information management system.

To validate the suitability of our proposed Mamba-YOLO-ML for deployment on embedded devices and mobile platforms, we developed a mulberry farm information management system. This system integrates equipment status monitoring, personnel management, and—most critically—real-time pest/disease detection functionality. The system interface is illustrated in Figure 12. The cross-platform React Native framework was adopted to bridge the Android–iOS divide, leveraging its standardized APIs to unify access to both operating systems’ native APIs and deep learning libraries. Prior to deployment, the model underwent INT8 quantization and pruning, followed by conversion to the ONNX format. Testing on an iPhone 12 (A14 processor, peak compute: 11 TOPS) demonstrated an average inference time of 95 ms per image (10.5 FPS), meeting real-time detection requirements.

## 4. Discussion

### 4.1. Analysis of Performance Improvement in the Enhanced Model Through Heatmap Comparison with the Original Model

The attention-focused mode utilizing heatmaps can better highlight the key areas of interest in the image at each layer of the model, while also providing a general understanding of the receptive field size of underlying blocks for each pixel. By recording the gradients of an image as it passes through each block layer of the model, we can generate corresponding heatmaps. In this experiment, we employed the Grad-CAM [55] algorithm to produce the relevant heatmaps.

We visualized the heatmaps of both the original Mamba-YOLO model and our improved Mamba-YOLO-ML model at the Stem layer and stages 1 through 4 of the backbone network, which sequentially correspond to our proposed Haar Stem and PMSS Block blocks.

**Figure 13:**
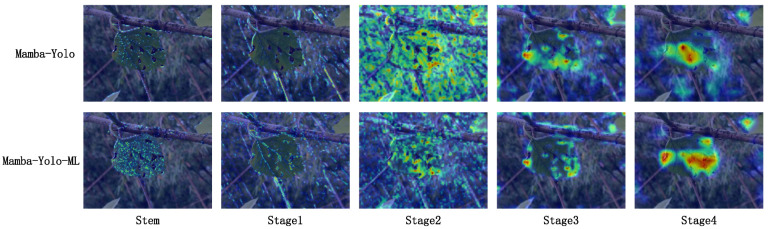
Heatmap comparison between the original Mamba-YOLO model and our improved Mamba-YOLO-ML model.

From Figure 13, we observe that in the Stem layer, the application of the Haar sampling algorithm enables better separation of high-frequency features (such as leaf vein textures) and low-frequency information (such as overall leaf contours and large homogeneous areas of mesophyll). Consequently, Mamba-YOLO-ML demonstrates superior capability in identifying mulberry leaf vein patterns and contours compared with the original model. At Stage 1, DSPy Block in the CE Block efficiently expands the receptive field with minimal computational cost, thereby enhancing the model’s low-channel information perception and enabling rapid detection and focus on disease characteristics. During Stage 2, while the original model’s receptive field has expanded sufficiently, it exhibits near-undifferentiated attention to all perceived features. In contrast, Mamba-YOLO-ML leverages the FG Block’s functionality to selectively filter features through channel-wise, point-wise, global, and local attention mechanisms operating on SS2D-processed global perception data. This allows precise disease localization with reduced interference from irrelevant features. A clear distinction emerges in Stages 3 and 4, where Mamba-YOLO-ML comprehensively captures all disease-related information, whereas the original model only attends to limited disease features along with erroneous characteristics. Thus, our heatmap experiments validate that Mamba-YOLO-ML achieves significantly improved disease-focused attention compared with the original model.

### 4.2. Generalizability Analysis in Classification Tasks

Due to the limited size of our dataset, which may not adequately represent broader generalization scenarios, there is a risk that specific models could exhibit biased advantages due to dataset quality issues. To address this, we incorporated the mulberry leaf disease classification dataset proposed by Nahiduzzaman et al. [16] and Salam et al. [19]. This original dataset comprises 1091 images across three categories: healthy, leaf rust, and leaf spot. The authors performed 5-fold cross-validation, with each fold containing 764 training images (augmented to 6000 samples), 218 test images, and 109 validation images. To evaluate the generalization capability of our models on external datasets, we conducted extensive validation on four top-performing architectures: Mamba-YOLO-ML, Mamba-YOLO, YOLOv9s, and YOLOv12s. Each model was evaluated three times using different random seeds (100 epochs per run), resulting in a total of 15-fold validation. This rigorous testing framework ensures robust assessment of cross-dataset generalization performance.

As shown in the experimental results Table 9, Mamba-YOLO-ML demonstrates consistent superiority in mean accuracy across all comparative models. Although the 95% confidence intervals exhibit substantial overlap, Mamba-YOLO-ML maintains notably narrower confidence bounds, indicating greater model stability with reduced performance fluctuations and higher experimental reliability. Furthermore, all comparative models yield *p*-values below 0.05, confirming statistically significant improvements of our enhanced model over both baseline approaches and YOLOv9/YOLOv12 architectures.

While our data augmentation strategy simulates diverse operational scenarios, we acknowledge two critical limitations: (1) performance degradation under extreme conditions (e.g., near-total darkness or camera overexposure), where model generalizability and detection accuracy may drop precipitously, and (2) practical deployment challenges concerning large-scale equipment installation, including hardware costs, infrastructure requirements, and technical personnel training. These issues represent key frontiers for future research in agricultural AI implementation.

### 4.3. Future Prospects of Mulberry Leaf Detection

Object classification and object detection have become core tools for agricultural pest and disease management due to their efficiency and ease of use. Classification models rapidly identify disease types by extracting global features, while detection models further achieve localization of symptomatic areas, providing a basis for precise pesticide application and disease severity assessment. For instance, this study realizes real-time detection of mulberry leaf diseases through improvements to the Mamba-YOLO algorithm, meeting the field requirements for low power consumption and high real-time performance. However, in practical operations, managers typically desire pixel-level detection accuracy to ensure no minor symptoms are overlooked.

Compared with classification and detection, object segmentation (e.g., semantic segmentation or instance segmentation) in agriculture remains in the exploratory stage, though small-scale studies exist, such as Liu et al. [56] on rice segmentation and Yang et al. [57] on tomato disease segmentation. Its limitations are mainly reflected in the following three aspects:

- High Data Annotation Costs: Object segmentation requires pixel-level labeling, whereas agricultural images often exhibit irregular and blurry disease/pest boundaries, making manual annotation labor-intensive. For example, fine-grained annotation of mulberry leaf disease spots demands expert-level knowledge, making large-scale dataset construction significantly more challenging than detection tasks.
- Computational Resource and Real-Time Bottlenecks: Segmentation models (e.g., U-Net [58] and Segnet [59]) have high parameter counts and slow inference speeds, making them difficult to deploy in field environments with stringent real-time requirements. Although lightweight segmentation networks have emerged, balancing accuracy and efficiency while achieving low-cost deployment on embedded devices remains a challenge.
- Complex Environmental Interference: Issues such as uneven lighting, leaf occlusion, and background noise in agricultural settings significantly degrade segmentation model performance. For instance, disease segmentation in overlapping mulberry leaf regions is prone to interference from adjacent healthy tissues, leading to increased missegmentation rates.

Over the next 10–20 years, artificial intelligence (AI) is poised to exert transformative and comprehensive impacts on agriculture. Farmers will be able to monitor entire farm operations remotely and confidently execute AI-driven decisions. However, we must remain vigilant about potential risks, such as AI’s unintended suppression of agricultural expertise and traditional knowledge, which could lead to a generational gap in skilled farmers. To mitigate this, we advocate for a dual-validation mechanism combining AI-assisted decision-making with human experience, alongside “digital preservation of traditional knowledge”—converting veteran farmers’ wisdom into AI-learnable unstructured data. Only by avoiding over-reliance on purely technical solutions can we ensure the sustainable and orderly development of agriculture.

## 5. Conclusions

This study proposes an optimized mulberry leaf disease detection model based on the Mamba-YOLO framework. Through phased module design, wavelet downsampling, and loss function improvements, we validate the research hypothesis of “enhancing multi-object detection accuracy and speed while reducing model complexity”. Experimental results demonstrate the superior performance of the improved model in detecting mulberry pests and diseases under natural environments, with the following key findings:

First, the PMSS Block enhances the local and global feature extraction capabilities of the SSM algorithm through the synergistic integration of the CE Block and the FG Block. The CE Block employs depthwise separable pyramid convolution, improving mAP50 for small lesion detection by 1.0% under low-channel conditions. The FG Block utilizes a multi-dimensional attention mechanism to filter redundant high-channel features, reducing model parameters by 6.7% while increasing computational efficiency by 10%.

Second, Haar Stem preserves high-frequency texture details of mulberry leaves through multi-resolution analysis, demonstrating its dual advantage of information retention and computational efficiency in agricultural image processing.

Third, the NWD loss function models bounding box similarity via Gaussian distribution, effectively addressing the sensitivity of traditional IoU to geometric deviations in small targets.

Comparative experiments show that the enhanced Mamba-YOLO model achieves leading comprehensive performance in detecting six categories of mulberry pests/diseases: mAP50 of 78.2% and mAP50:95 of 59.9%, significantly outperforming YOLOv8n (74.8%) and YOLOv12s (76.7%). The improved model reduces parameters to 5.6 M (6.7% compression from the baseline 6.0M), with its lightweight characteristics making it suitable for mobile diagnostic tools, providing technical support for precision pesticide application and ecological monitoring in mulberry plantations.

This research establishes an efficient algorithmic framework for intelligent mulberry disease management. Future work will explore cross-crop generalization capabilities and integrate semantic segmentation techniques for lesion area quantification, further advancing precision agriculture and sustainable development.

## Figures and Tables

**Table 1:** Distribution of anchor boxes across different categories in the dataset.

Category ID	Name	Number of Boxes
0	Anthracnose	2676
1	Brown Spot	961
2	Bacterial Blight	545
3	Healthy	3641
4	Aulacophora Femoralis	172
5	Chrysochus	199
Total		8194

**Table 2:** PMSS Block stage allocation across network stages.

	Stage1	Stage2	Stage3	Stage4	PAFPN
Low Channel	DSPy	LS	LS	LS	LS
High Channel	RG	MAGG	MAGG	MAGG	MAGG

**Table 3:** Performance comparison of different object detection models on the mulberry leaf disease dataset.

Model (%)	Precision (%)	Recall (%)	mAP_50_ (%)	mAP_50:95_ (%)	Params (M)	GFLOPs	Size (MB)
Faster R-CNN	76.6	61.5	70.1	43.4	28.3	135.0	220.0
SSD	73.0	61.7	65.7	34.5	24.4	30.7	186.0
YOLOv8s	81.8	68.8	75.8	54.7	9.8	23.4	19.0
YOLOv9s	80.5	71.5	77.1	59.4	6.1	22.1	12.7
YOLOv10s	79.2	68.7	75.8	56.1	8.0	24.5	15.7
YOLOv11s	82.2	68.6	75.4	55.9	9.4	21.3	18.2
YOLOv12s	83.6	67.9	76.7	57.6	9.3	21.5	18.0
RT-DETR-R18	76.9	70.6	72.9	53.5	19.9	52.8	38.5
Mamba-YOLO	78.0	71.0	77.1	58.8	6.0	13.6	11.7
Mamba-YOLO-ML	80.7	72.1	78.2	59.9	5.6	13.4	11.1

**Table 4:** Ablation study of Mamba-YOLO-ML components.

PMSS Block		Haar Stem	NWD Loss	Precision	Recall	mAP_50_	mAP_50:95_	Params	GFLOPS
CE Block	FG Block			(%)	(%)	(%)	(%)	(M)	
				78.0	71.0	77.1	58.8	6.0	13.6
✓				78.9	72.1	78.1	58.7	6.0	14.2
	✓			78.3	70.8	77.4	58.9	5.6	12.9
		✓		77.5	71.5	77.7	59.4	6.0	13.5
			✓	83.6	67.5	77.3	59.0	6.0	13.6
✓	✓			79.9	70.6	77.5	59.3	5.6	13.4
✓	✓	✓		80.9	71.9	78.0	59.0	5.6	13.4
✓	✓	✓	✓	80.7	72.1	78.2	59.9	5.6	13.4

**Table 5:** Ablation study on DSPy Block design variants.

Test	Precision	Recall	mAP_50_	mAP mAP_50:95_	Params	GFLOPS
	(%)	(%)	(%)	(%)	(M)	
DSPy Block	78.9	72.1	78.1	58.7	6.0	14.2
DSPy w/cat	80.8	71.5	77.5	58.9	7.0	18.9
DSPy w/o res	83.6	68.6	77.6	59.5	6.0	14.2

**Table 6:** Ablation study on MAGG Block design variants.

Test	Precision	Recall	mAP_50_	mAP_50:95_
	(%)	(%)	(%)	(%)
MAGG Block	78.3	70.8	77.4	58.9
MAGG w/stack	78.2	70.7	77.0	59.0
MAGG w/one selection	82.6	67.6	76.8	59.0

**Table 7:** Ablation study on Haar wavelet position.

Test	Precision	Recall	mAP_50_	mAP_50:95_	Params	GFLOPS
	(%)	(%)	(%)	(%)	(M)	
Haar on Stem	**77.5**	71.5	**77.7**	**59.4**	**6.0**	**13.5**
Haar on Downsampling	76.4	**72.1**	77.3	58.8	**6.0**	13.6

**Table 8:** Ablation study on Loss Fuction.

Test	Precision	Recall	mAP_50_	mAP_50:95_
	(%)	(%)	(%)	(%)
NWD	80.7	72.1	78.2	59.9
CIOU	80.9	71.9	78.0	59.0
Focal	77.8	71.9	77.4	59.0
EIOU	79.4	69.7	76.4	59.3
SIOU	77.6	70.9	77.0	59.4
WIOU	80.5	70.2	77.0	58.8

**Table 9:** Classification performance comparison on external mulberry dataset.

Model	Mean Accuracy (%)	95% CI (%)	*p*-Value (vs. Mamba-YOLO-ML)
Mamba-YOLO-ML	94.80 ± 4.94	[92.07, 97.53]	-
Mamba-YOLO	93.03 ± 5.60	[89.93, 96.13]	0.0030
YOLOv9s	93.70 ± 5.20	[90.82, 96.58]	0.0391
YOLOv12s	92.84 ± 6.15	[89.44, 96.25]	0.0038

## Data Availability

Data will be made available on request.
